# The 24-hour urinary cortisol in post-traumatic stress disorder: A meta-analysis

**DOI:** 10.1371/journal.pone.0227560

**Published:** 2020-01-09

**Authors:** Xiongfeng Pan, Atipatsa C. Kaminga, Shi Wu Wen, Zhipeng Wang, Xiaoli Wu, Aizhong Liu

**Affiliations:** 1 Department of Epidemiology and Health Statistics, Xiangya School of Public Health, Central South University, Changsha, China; 2 Department of Mathematics and Statistics, Mzuzu University, Mzuzu, Malawi; 3 Department of Obstetrics and Gynaecology, University of Ottawa, Ottawa, Ontario, Canada; 4 Ottawa Hospital Research Institute, Ottawa, Ontario, Canada; Universita degli Studi di Milano-Bicocca, ITALY

## Abstract

**Objective:**

Previous studies found inconsistent results on the relationship between post-traumatic stress disorder (PTSD) and concentrations of 24-hour (24-h) urinary cortisol. This study performed a systematic review and meta-analysis to summarize previous findings on this relationship.

**Methods:**

We searched in the databases of Web of Science, PubMed, Embase, and Psyc-ARTICLES for articles published before September 2018. We used the random-effects model with restricted maximum-likelihood estimator to synthesize the effect sizes by calculating the standardized mean difference (SMD) and assessing its significance.

**Results:**

Six hundred and nineteen articles were identified from the preceding databases and 20 of them were included in the meta-analysis. Lower concentrations of 24-h urinary cortisol were observed in patients with PTSD when compared with the controls (SMD = -0.49, 95%CI [-0.91; -0.07], *p =* 0.02). Subgroup analysis revealed that the concentrations of 24-h urinary cortisol were lower in PTSD patients than in the controls for studies that included female participants or studies that included participants from the United States of America.

**Conclusions:**

Overall, decreased levels of 24-h urinary cortisol were linked with the pathophysiology of PTSD. Nonetheless, more studies should be conducted to validate the molecular underpinnings of urine cortisol degeneration in PTSD.

## Introduction

As a complex multifactor psychiatric disorder, post-traumatic stress disorder (PTSD) is triggered by unusual shocks or catastrophic events [1], and its syndromes are characterized by arousal, avoidance, and intrusion [2], which would cause a greater risk for debilitating physical and mental health outcomes [3]. Therefore, PTSD can negatively affect an individual’s Quality of Life and work performance [4,5]. In addition, some studies indicated that PTSD was directly responsible for suicidality [6]. Thus, PTSD is an important global public health issue that needs effective prevention and control measures [7,8].

Along with genetic predisposition, and environmental factors [9], PTSD could cause an effect on people’s psychological state, and neurobiological state such as affecting the hypothalamic–pituitary–adrenal (HPA) axis regulation [10,11]. For example, during acute stress, the HPA axis is activated, and the hypothalamus secretes corticotropin-releasing hormone under the influence of serotonin from the amygdala [12]. Then, corticotropin-releasing hormone stimulates the pituitary gland to release adrenocorticotropic hormone, which causes the adrenal cortex to produce cortisol, whose influence can block many immune reactions, neuronal defensive and metabolic mechanisms [13]. These physiological processes allow an individual to retain more energy that can be mobilised to cope adequately with the stressor. However, in chronic stress, HPA-axis becomes inactive, leading to a decrease in cortisol secretion [14]. Among several techniques now used to assess HPA-axis functioning, the function of cortisol measurement has come into notice. [15,16]. It has been suggested that cortisol could be used as a biomarker in PTSD screening [17]. However, up to now, little is known about the biological mechanisms behind this differential risk [18].

There are various biological specimens such as plasma, serum, saliva, hair, cerebrospinal fluid and urine that could be used to measure cortisol [19–21]. Nevertheless, because of their noninvasive nature, saliva and urine have always been chosen as the preferred sources of samples for cortisol measurement [22–24]. It is generally believed that 24-h urinary cortisol levels provide an integrated measure that is more trustworthy than that obtained from random salivary samples [25].

In future, it is expected that 24-h urinary cortisol could be a quick biomarker assay to assist in screening patients for PTSD. Studies on 24-h urinary cortisol alterations in screening PTSD have so far yielded inconsistent results both in direction and magnitude of hormonal changes. Although most studies found that PTSD was associated with lower 24-h urinary cortisol output, some earlier studies showed contrary results [26,27].

There had been no meta-analysis primarily examining 24-h urinary cortisol as a biomarker for PTSD screening [28]. However, 24-h urinary cortisol had been used in subgroup analysis of some previous meta-analyses but sample sizes were small and results were not significant [29]. Therefore, the aim of this study was to conduct the first comprehensive systematic review and meta-analysis on 24-h urinary cortisol alterations in PTSD. Also, regression and subgroup analysis were used to investigate sources of heterogeneity among studies.

## Methods

### Data sources and search strategy

The comprehensive literature search for relevant studies followed the guidance by the Preferred Reporting Items for Systematic Reviews and Meta-Analyses (PRISMA) criteria (last search updated in September 2018) [30]. This systematic review and meta-analysis is registered, and the full protocol was uploaded to the International Prospective Register of Systematic Reviews website (CRD42018109958).

The search for relevant articles was restricted to articles published in the English language. This was performed in the following four electronic databases: Web of Science, PubMed, Embase, and Psyc-ARTICLES. The search strategy was designed in consultation with experienced librarians. For example, a search strategy in the database of Embase was structured as follows using keywords (search terms): ('urinary free cortisol':ab,ti OR 'urinary cortisol':ab,ti OR 'cortisol in urine':ab,ti OR 'urine cortisol':ab,ti OR 'glucocorticoids in urine':ab,ti OR 'urine glucocorticoid':ab,ti OR 'steroid hormones in urine':ab,ti OR 'urine steroid':ab,ti OR 'urine corticosteroid':ab,ti OR 'urinary cortisol':ab,ti) and ('ptsd':ab,ti OR 'posttraumatic stress disorder':ab,ti). In addition, hand searching was conducted in the lists of references of the retrieved articles by XP and AC. Any inconsistencies between them were resolved by group discussion and consensus with a third party AL.

### Eligibility criteria

A study eligible for this meta-analysis had to meet the following inclusion criteria: (1) the study had to be a case control study, which included a control group and PTSD cases, (2) the study reported the mean and standard deviation (SD) of the 24-h urinary cortisol levels, or these could be provided by the authors upon request, (3) the study assessed PTSD cases simultaneously with the assessment of the 24-h urinary cortisol levels, (4) the study was published in English, and (5) the study reported the diagnostic criteria for PTSD. In addition, studies were excluded if they (1) were review articles or case reports; (2) studied PTSD in combination with HPA axis disorder disease, or studied PTSD in patients with other mental illnesses, who used psychotropic medication or other medications which could influence the HPA axis and cortisol concentrations, and (3) studied non-humans or were vitro experiments or animal research.

### Data extraction

Two researchers [XW and ZW] independently screened and selected eligible articles [31]. A third party was involved in consultations to make the final decision in the event of disagreements [AL]. Moreover, the grey literatures (non-published literatures) were excluded from our study [32].

In relation to the purpose of this study, the following information were extracted from the eligible studies by two independent investigators [AC and XW]: (1) name of first author, and publication year; (2) geographical area of the study; (3) characteristics of PTSD participants such as trauma type, age (mean, SD), gender distribution, and body-mass index (BMI) (mean, SD); (4) sample characteristics such as sample size, and concentrations of 24-h urinary cortisol (mean, SD); (5) PTSD assessment method; and (6) 24-h urinary cortisol collection and assay methods, intra-assay variation, inter-assay variation, storage temperatures and sensitivity. All the extracted data were organized in EpiData 3.0 and saved in Excel.

### Quality evaluation

The Newcastle-Ottawa Quality Assessment Scale (NOS) was used to assess the quality of the eligible studies [33]. Therefore, each eligible study was evaluated based on the three broad perspectives: (1) Selection; (2) Comparability; and (3) Outcome. Two investigators [XP and AC] independently assessed and graded the eligible studies. Any inconsistencies between them were resolved by group discussion with a third party [AL]. According to the pre-specified criteria of this scale, studies scoring 7–9, 4–6, and 0–3 points were graded, respectively, as high, moderate, and low quality.

### Statistical analysis

All analyses were conducted in R software (version R i386 3.4.2). Accordingly, meta-analysis was carried out in the package, meta, and meta-regression analysis was performed in the package, metafor. The standardized mean difference (SMD) of the 24-h urinary cortisol levels between the PTSD and the control groups was calculated using Cohen’s d [34]. Moreover, by using restricted maximum-likelihood estimator to synthesize the effect sizes reported in the eligible studies, random-effects models were fitted. The Q-test was carried out to examine whether there was heterogeneity in the results from the eligible studies. This heterogeneity was quantified using the I^2^ statistic (I^2^ = 0% indicates no heterogeneity and I^2^ = 100% indicates maximal heterogeneity) [34]. In order to explore sources of heterogeneity between the eligible studies, subgroup analysis was performed with respect to controls type. Specifically, the following subgroups were used for subgroup analyses in this study: study country (USA or not USA); controls type (trauma-exposed controls (TC) or non-trauma-exposed controls (NTC)); and assayed methods (radioimmunoassay (RIA) or other). Besides, the fact that gender-specific 24-h urinary cortisol concentration data were not provided by the eligible studies, an eligible study with over 50% proportion of women in the total sample was defined as examining female subjects. In this way, subgroup analysis in relation to gender was performed. In addition, sensitivity analysis was performed to prove the stability of the results. Also, potential publication bias was assessed using the symmetry of a funnel plot, whose interpretation was verified by the Egger’s linear regression test [35].

Finally, in all the statistical tests, the level of significance was set at the 5%, and all tests were two-sided. Furthermore, other sources of heterogeneity were explored using meta-regression analysis. Therefore, the following categorical variables were considered for the meta-regression model: country (USA = 1, other = 0), trauma type (combat = 1, other = 0), controls type (TC = 1, NTC = 0), gender (male = 1, female = 0), PTSD assessment (Diagnostic and Statistical Manual of Mental Disorders 4th edition (DSM-IV) = 1, other = 0), assayed method (RIA = 1, other = 0), frozen samples (report = 1, unreported = 0). In addition, the following continuous variables were considered for the meta-regression model: age, study quality and BMI.

## Results

### Literature search and eligible studies

**Fig 1:**
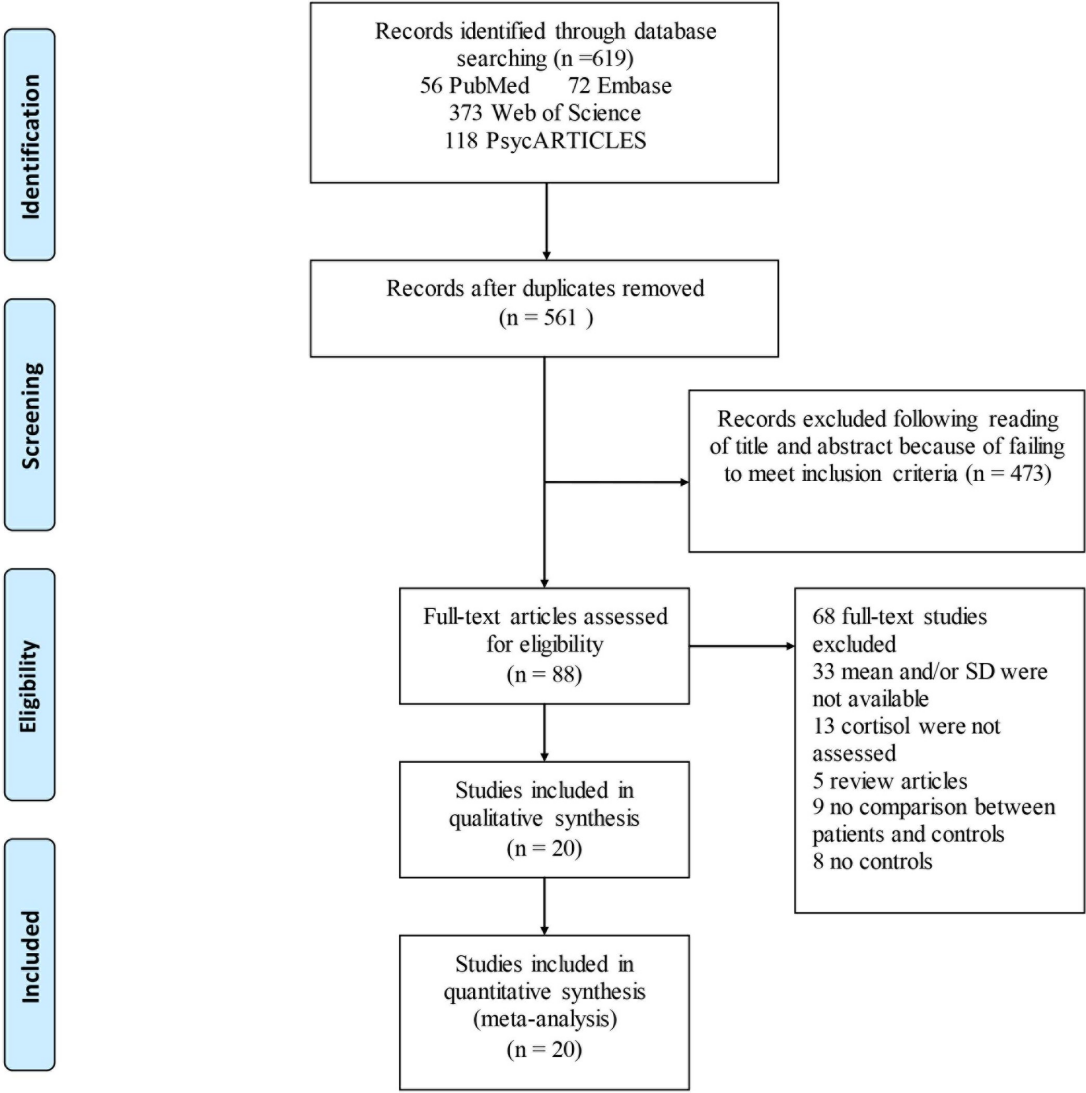
Flow chart of study selection. Showing the process by which relevant studies were retrieved from the databases, assessed, and selected, or excluded. Preferred reporting items for systematic reviews and meta-analyses (PRISMA) diagram for study search.

The search of literature yielded 619 relevant articles from Web of Science (373), PubMed (56), Embase (72), and PsycARTICLES (118). After that, 58 duplicates were deleted, leaving a total of 561 relevant articles. The abstracts of these articles were then reviewed to assess their eligibility. Following this assessment, 473 articles did not meet the inclusion criteria, hence they were excluded. Furthermore, full texts of the 88 articles were reviewed and this resulted in the exclusion of 68 articles. In the end, 20 eligible articles met the inclusion criteria and were included in the final meta-analysis (Fig 1).

### Characteristics of the articles

Table 1 presents study characteristics of the 20 eligible studies. Most articles reported controls type, 24-h urinary cortisol collection time, 24-h urinary cortisol collection and assay methods, characteristics of PTSD participants (trauma type, age (mean, SD), BMI, and gender distribution), inter-assay variation, 24-h urinary cortisol intra-assay variation, sensitivity and storage temperature. Three studies were judged to be of high-quality (total score ≥7), 15 of moderate quality, and 2 of low quality (Table 1).

**Table 1:** Characteristics of studies included in the meta-analysis.

Study		BMI	Country	Trauma type	Controls	Female	Quality evaluation	Mean Age	PTSD Assessment	Methods	Interassay variation	Intraassay variation	Frozen
**(Bader et al., 2014)**	[46]	24.7±4.1	USA, Canada	Holocaust	TC	26(61.9%)	6	47.6±7.5	DSM-IV CAPS	RIA	NR	NR	NR
**(Baker et al., 1999)**	[47]	25.9±4.1	USA	Combat	NTC	0(0%)	7	41.4±8.2	DSM-III-R (SCID)	RIA	0.12	0.07	0°C
**(Bierer et al., 2006)**	[48]	NR	USA	Attack	TC	25(59.5%)	5	42.1±10.1	DSM-IV(SCID)	RIA	NR	NR	NR
**(De Bellis et al., 1999)**	[49]	1.29±0.22	USA	Sexual abuse	NTC	8(80%)	6	10.4±1.4	DSM-III-R AxisI	RIA	NR	NR	-80°C
**(Delahanty et al., 2000)**	[50]	NR	USA	Vehicle accident	TC	36(36.4%)	6	37.3±17.7	DSM-IV(SCID)	Flourescent polarization immunoassay	NR	NR	0°C
**(Lemieux et al., 1995)**	[51]	NR	USA	Sexual abuse	NTC	28(100%)	6	35.3±6.3	DSM-III-R	RIA	0.06	0.04	NR
**(Lemieux et al., 2008)**	[52]	NR	USA	Maltreatment	NTC	72(100%)	5	30.3±6.4	DSM-III-R	Immunofluorescence	NR	NR	NR
**(McFarlane et al., 2011)**	[53]	NR	Australia	Traumatic accident	TC	12(25%)	8	34±12.7	DSM-IV CAPS	RIA	0.08	0.05	-20°C
**(Masoudzadeh et al., 2013)**	[54]	NR	Iran	Combat	NTC	0(0%)	3	41.9±7.6	DSM–IV-IR	NR	NR	NR	NR
**(Otte et al., 2005)**	[26]	27±5	USA	Combat	NTC	0(0%)	4	49±7	DSM-IV CAPS	RIA	NR	NR	NR
**(Pitman et al., 1990)**	[55]	NR	USA	Combat	NTC	0(0%)	6	40.9±6.1	DSM-III-R	RIA	0.07	0.07	-70°C
**(Rasmusson et al., 2001)**	[56]	NR	USA	Mixed trauma	NTC	12(100%)	7	37.3±2.1	DSM-IV CAPS	RIA	0.06	0.03	0°C
**(Simeon et al., 2007)**	[57]	NR	USA	Mixed trauma	TC	15(48%)	5	31.2±11.6	DSM-IV CAPS	HPLC	NR	NR	0°C
**(Wheler et al., 2006)**	[27]	NR	USA	Mixed trauma	NTC	7(70%)	3	NR	DSM-IV CAPS	GC-MS	NR	NR	NR
**(Wingenfeid et al., 2015)**	[58]	NR	USA	Combat	TC	25(12.6%)	5	57.4±11.0	DSM-IV CAPS	HPLC	NR	NR	0°C
**(Yehuda et al., 2008)**	[59]	26.0±4.4	USA	Mixed trauma	NTC	16(69.6%)	5	50.4±7.3	DSM-IV CAPS	NR	NR	NR	NR
**(Yehuda et al., 2000)**	[60]	NR	USA	Mixed trauma	NTC	29(82.9%)	6	40.9±6.4	DSM-IV CAPS	RIA	NR	NR	0°C
**(Yehuda et al., 2001)**	[61]	NR	USA	Mixed trauma	TC	31(56.4%)	6	40.9±7.6	DSM-IV CAPS	RIA	NR	NR	0°C
**(Yehuda et al., 2007)**	[62]	NR	USA	Mixed trauma	TC	0(0%)	6	60.6±7.0	DSM-IV CAPS	RIA	0.07	0.04	0°C
**(Yehuda et al., 2009)**	[63]	26.3±0.6	USA	Mixed trauma	TC	0(0%)	6	73.1±1.0	DSM-IV CAPS	RIA	0.10	0.10	0°C

TC, trauma-exposed controls; NTC, non-trauma-exposed Controls; RIA, radioimmunoassay; GC-MS, Gas chromatography-mass spectrometry; HPLC, High performance liquid chromatography-tandem mass spectrometry; CAPS, clinician-administered PTSD scale; NR, not report; USA, United States of America; DSM, Diagnostic and Statistical Manual of Mental Disorders.

### 24-h urinary cortisol overall comparison

**Fig 2:**
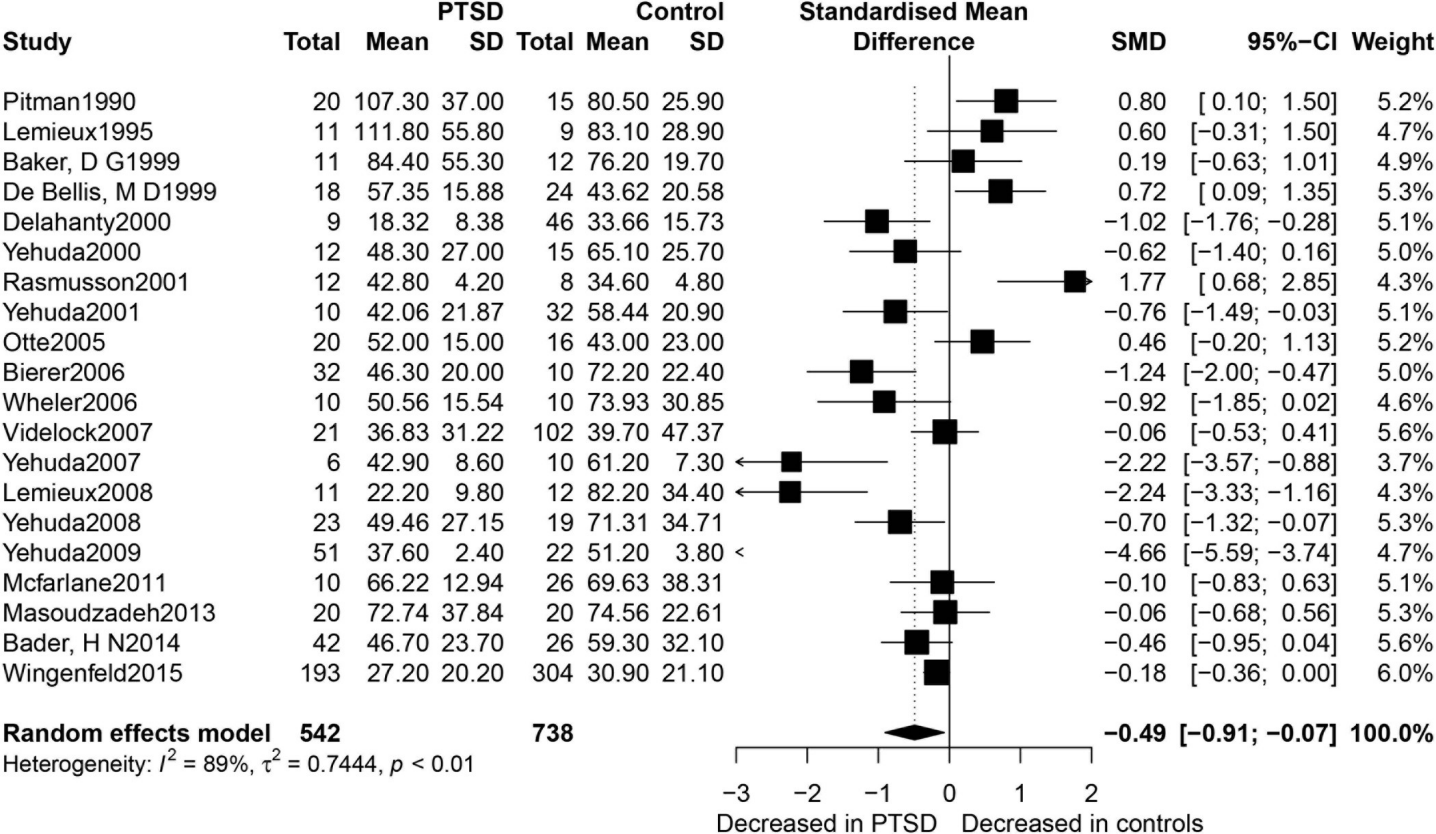
Forest plot of 24-h urinary cortisol between PTSD participants and controls. Study effect sizes of urinary cortisol concentrations differences between PTSD and controls. Each data marker represents a study, and the size of the data marker is proportional to the total number of individuals in that study. The summary effect size for each urinary cortisol concentration is denoted by a diamond. PTSD, post traumatic stress disorder; SMD, standardised mean difference.

Fig 2 presents a forest plot for the SMD constructed from a random-effects model of the concentrations of the 24-h urinary cortisol between PTSD patients and controls in the 20 eligible studies. Lower concentrations of 24-h urinary cortisol were found in patients with PTSD than in the controls (SMD = -0.49, 95%CI [-0.91; -0.07], *p =* 0.021), but with considerable heterogeneity (*I*^*2*^ = 89%, and *p<* 0.0001 for the Q-test).

### Subgroup analyses

Table 2 presents the subgroup analyses for 24-h urinary cortisol levels between PTSD patients and controls. For studies conducted in the USA, patients with PTSD had highly significant lower concentrations of 24-h urinary cortisol than the controls (SMD = -0.55, 95%CI: [-1.05; -0.05], *p =* 0.031), while for studies conducted elsewhere there was no significant difference. Additionally, subgroup analysis with respect to gender indicated that females with PTSD had lower concentrations of 24-h urinary cortisol than female controls (SMD = -0.58 95%CI: [-1.09; -0.07], *p = 0*.*026*), but no such difference was observed between males with PTSD and male controls. Besides, gender significantly explained a large amount of the overall heterogeneity, but still with residual heterogeneity (*I*^*2*^ = 11.3%). Furthermore, subgroup analysis by controls type showed significant lower concentrations of 24-h urinary cortisol in patients with PTSD than in the trauma exposed controls (SMD = -1.10, 95% CI: [-1.77; -0.43], *p =* 0.001), while there was no significant difference between PTSD patients and the non-trauma-exposed controls. In the subgroup analysis according to assay methods, the concentrations of 24-h urinary cortisol were significantly lower in patients with PTSD than in the controls when radioimmunoassay (RIA) was used (SMD = -0.51, 95%CI [-0.97; -0.04], *p =* 0.032), while no difference was found when other assay methods were used.

**Table 2:** Subgroup analysis.

	N	SMD (95% CI)	Z value	p value	Heterogeneity		
					Q statistic (DF; p value)	τ^2^	I^2^
**All**	20	-0.49 [-0.91; -0.07]	-2.30	0.021	168.10 19 < 0.0001	0.74	88.70%
**Study country**							
**USA**	17	-0.55 [-1.05; -0.05]	-2.16	0.031	166.87 16 < 0.0001	0.94	90.40%
**Not USA**	3	-0.26 [-0.60; 0.08]	-1.48	0.140	1.21 2 0.5458	0.00	0.00%
**Gender**							
**Female**	10	-0.58 [-1.09; -0.07]	-2.23	0.026	8.69 9 0.5071	0.03	11.30%
**Male**	10	-0.43 [-1.08; 0.23]	-1.28	0.200	119.25 9 < 0.0001	0.97	92.50%
**Controls type**							
**TC**	9	-1.10 [-1.77; -0.43]	-3.21	0.001	105.39 8 < 0.0001	0.91	92.40%
**NTC**	11	0.016 [-0.51; 0.54]	0.06	0.952	51.07 10 < 0.0001	0.62	80.40%
**Assayed methods**							
**RIA**	18	-0.51 [-0.97; -0.04]	-2.14	0.032	165.89 17 < 0.0001	0.84	89.80%
**Other**	2	-0.37 [-1.00; 0.25]	-1.17	0.241	2.02 1 0.1555	0.10	50.40%

PTSD, post traumatic stress disorder; SMD, standardised mean difference; DF, degrees of freedom; TC, trauma-exposed controls; NTC, non-trauma-exposed controls; RIA, radioimmunoassay; USA, United States of America.

### Meta-regression analyses

Table 3 presents the results of meta-regression analysis. The results were not significant in relation to country, controls type, gender, assayed method, frozen samples, study quality and BMI. However, after introducing trauma type (b = 2.7401, 95%CI 1.1920; 4.2881, *p =* 0.0005), PTSD assessment (b = 1.2768, 95%CI 0.1214; 2.4321, *p =* 0.0303) and age (b = -0.0831, 95%CI -0.1380;-0.0282, *p =* 0.0030) in the meta-regression analysis model, the heterogeneous sources could be explained, and the difference was significant.

**Table 3:** Separate univariate meta-regression model of 24-h urinary cortisol in PTSD.

	Estimate	Standard error	Z value	p value	95% CI	
**intrcpt**	-0.0053	1.6385	-0.0033	0.9974	-3.2168	3.2061
**Country**	0.2804	0.6661	0.4209	0.6738	-1.0251	1.5859
**Trauma type**	2.7401	0.7898	3.4692	**0.0005**	1.1920	4.2881
**Controls type**	-0.1990	0.6375	-0.3121	0.7550	-1.4485	1.0505
**Gender**	-0.6820	0.7575	-0.9003	0.3679	-2.1665	0.8026
**PTSD Assessment**	1.2768	0.5895	2.1660	**0.0303**	0.1214	2.4321
**Assayed method**	0.5837	0.6771	0.8621	0.3886	-0.7433	1.9107
**Frozen samples**	-1.1668	0.8209	-1.4214	0.1552	-2.7757	0.4421
**BMI**	-0.2769	0.5085	-0.5446	0.5860	-1.2735	0.7197
**Age**	-0.0831	0.0280	-2.9686	**0.0030**	-0.1380	-0.0282
**Study quality**	0.3754	0.2794	1.3434	0.1791	-0.1723	0.9230

PTSD, post-traumatic stress disorder; BMI, Body Mass Index.

### Sensitivity and bias analysis

**Fig 3:**
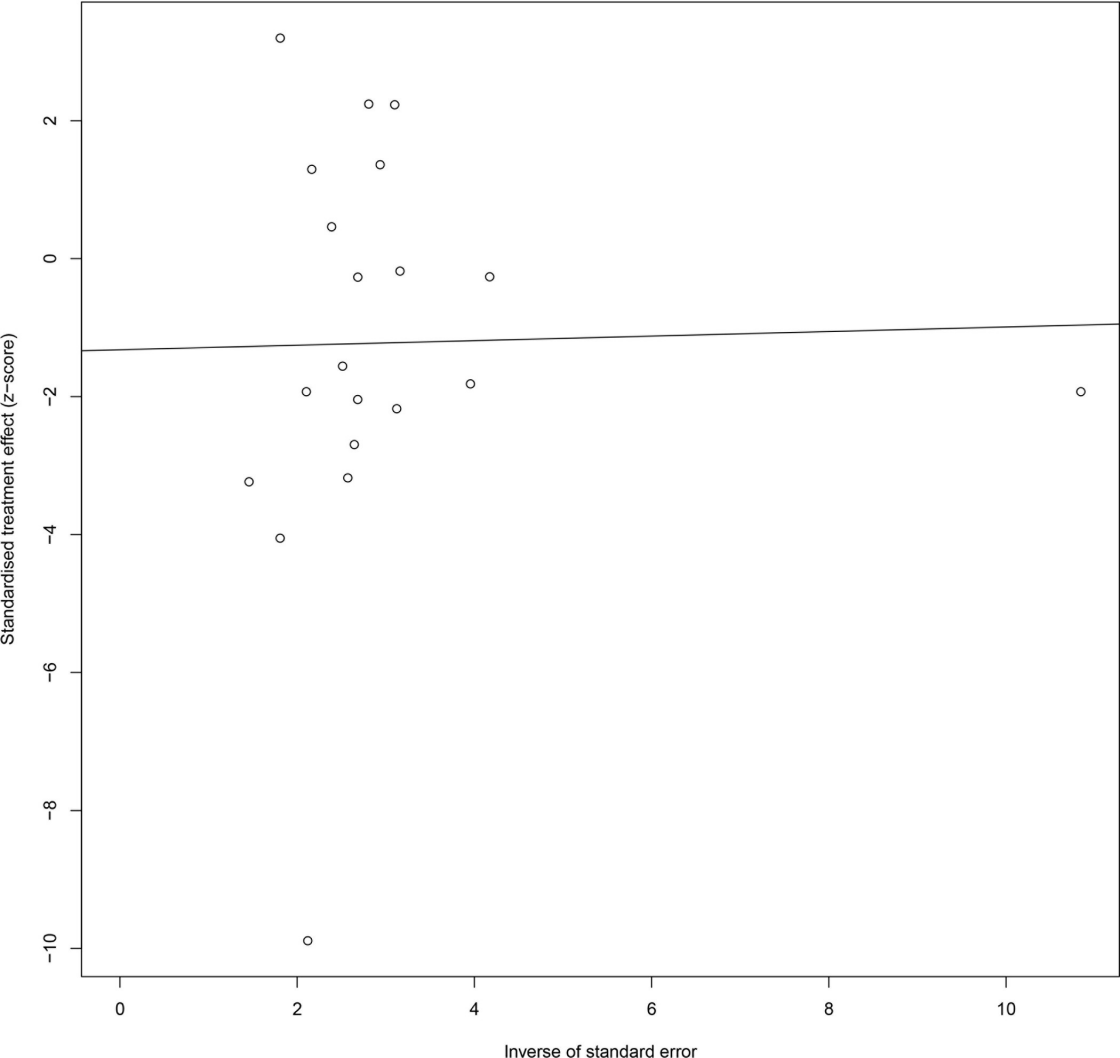
Egger funnel plots of 24-h urinary cortisol. Egger funnel plots to assess publication bias. Plots show study size as a function of effect size for studies included in the meta-analysis. The dots represent each study.

There was little change in the SMD and corresponding 95% CI when studies were excluded one at a time, indicating low sensitivity of this meta-analysis. Also, no asymmetry was observed in the shape of the Egger’s funnel plot, and the p value (0.31) of the Egger’s test was not significant (Fig 3), implying small chance of publication bias [35].

## Discussion

According to the best of our knowledge, this is the first meta-analysis comparing the 24-h urinary cortisol levels data between PTSD patients and the controls. In order to obtain relatively larger sample size, we expanded the scope of article searching in the online electronic databases, which brings 20 eligible studies with a total of 543 participants with PTSD and 738 controls. In the overall study sample, concentrations of 24-h urinary cortisol were lower in patients with PTSD than in controls. Therefore, the conclusions from this study are more comparative and persuasive compared with the previous studies. Besides, different from the preceding studies, this study only concerns with 24-h urinary cortisol concentration levels when studying its connection with PTSD.

In addition to data on the differences in the 24-h urinary cortisol levels between PTSD patients and the controls, this study also analyzed almost all factors that may affect 24-hour urinary cortisol levels, including subgroup analyses. In particular, a lower 24-h urinary cortisol levels was found in PTSD patients as compared to the controls. However, the heterogeneity was quite large; suggesting that there may be differences based on some certain subgroups. It is worth noting that our study had significant heterogeneity among the studies, which is not surprising considering the differences between research variations, such as participant characteristics. The previous meta-analyses only included the 24-h urinary cortisol levels data in subgroup analysis, but no significant differences were found between PTSD patients and the controls because of the limited sample size of the studies used [29].

Although previous studies had obtained consistent findings that plasma cortisol levels in PTSD patients was lower than that of the controls [36], they did not find significant difference in the 24-h urinary cortisol levels between PTSD patients and the controls [37]. In addition, these studies did not take into account the sample source when measuring cortisol concentration levels, such as plasma/serum, saliva or urine samples. Yet, it is generally known that the level of cortisol concentration between sufficient samples varies with different sample sources. Therefore, without taking the preceding factors into account, results of a meta-analysis on the critical relationship between cortisol concentration levels and PTSD could be compromised. Furthermore, subgroup analysis indicated that the 24-h urinary cortisol levels were lower in the PTSD patients than in the controls for studies conducted in the United States of America, whereas no difference was found in the studies conducted elsewhere. In general, females are twice as likely as males to develop PTSD; perhaps because females appear to have a more sensitized HPA axis with lower overall plasma cortisol as males, which are especially of interest with respect to cortisol, which interacts with sex hormones [38].

Accordingly, the findings of this study suggested that females with PTSD had lower levels of 24-h urinary cortisol than female controls, whereas there was no significant difference of the same between males with PTSD and male controls. However, up to now, little is known about the biological mechanisms behind the preceding association between the 24-h urinary cortisol levels and PTSD in females. In spite of that, this association could explain why women are more vulnerable than men to the development of post-trauma symptoms, and take longer than men to recover from them [39]. Also, this study found that the 24-h urinary cortisol levels were lower in the PTSD patients than in the controls in studies that used trauma-exposed controls, while no significant group difference of the same was found for studies that used non-trauma-exposed controls.

When 24-h urinary cortisol collection method [40], and storage methods of 24-h urinary cortisol has be unified [41]. The 24-h urinary cortisol could be a quick biomarker assay to assist in screening a possibility for screening a lot of people with PTSD [10,42]. Furthermore, meta-regression analysis results suggested that trauma type may be a source of heterogeneity, implying that levels of the 24-h urinary cortisol in PTSD caused by combat, for example, are different from levels of the same in PTSD caused by other trauma types. Nevertheless, the eligible studies did not provide information regarding severity of PTSD; hence this was not taken into account in this study. Therefore, the preceding result should be interpreted with caution. As regards studies using DSM-IV method to diagnose PTSD, the 24-h urinary cortisol levels were significantly different between PTSD patients and controls, while no group difference of the same was found when other diagnostic methods were used. This observation supports the importance of using valid diagnostic tools for PTSD assessment [43].

Also, age in this study explained some of the heterogeneity. This is consistent with results of a previous study, suggesting that the increased risk for developing PTSD is associated with lower concentrations of cortisol in both adults and children [44]. That is, during childhood, trauma exposure may have greater potential of devastating HPA, disrupting brain maturation and affecting the development of the frontal cortex, leading to long-term changes in the HPA response [45]. This may be related to prefrontal cortex development, corpus callosum myelination, and synaptic elimination in the developmental traumatology models. In addition, the low activity of the HPA in adults indicates a greater cumulative lifetime of trauma exposure, adaptation of the HPA axis, and the risk of developing PTSD [44].

### Limitations and strengths

However, this study has several limitations. First, due to the limited sample size, the effects of race, age and stressor patterns were not examined. Second, we were unable to use all studies in the subgroup analyses because not all eligible studies reported the subgroups of interest. Third, some studies did not report the use of urine standardization to control variability associated with urine dilution. Lastly, we included only articles published in English and excluded grey literatures. Therefore, this meta-analysis is prone to selection bias.

## Conclusions

Despite the preceding limitations, the results of this meta-analysis provide convincing evidence that lower concentrations of 24-h urinary cortisol may be associated with PTSD. However, it is worth noting that numerous often-overlooked factors may have a confounding influence on the concentrations of 24-h urinary cortisol. Therefore, future studies should elucidate whether low 24-h urinary cortisol is related to trauma type, age, or PTSD assessment methods.

## Supporting information

S1 Table Search strategies. Details of search strategy.
(DOC)

S2 Table PRISMA checklist. (DOC)

## References

[1] Franklin CL, Raines AM, Chambliss JL, Walton JL and Maieritsch KP. Examining various subthreshold definitions of PTSD using the Clinician Administered PTSD Scale for DSM-5. J Affect Disord.2018; 234: 256–260. 10.1016/j.jad.2018.03.001 29550742

[2] Feduccia AA and Mithoefer MC. MDMA-assisted psychotherapy for PTSD: Are memory reconsolidation and fear extinction underlying mechanisms? Prog Neuropsychopharmacol Biol Psychiatry.2018; 84: 221–228. 10.1016/j.pnpbp.2018.03.003 29524515

[3] Ke J, Zhang L, Qi R, Xu Q, Zhong Y and Liu T et al Typhoon-Related Post-Traumatic Stress Disorder and Trauma Might Lead to Functional Integration Abnormalities in Intra- and Inter-Resting State Networks: a Resting-State Fmri Independent Component Analysis. Cellular Physiology and Biochemistry.2018; 48: 99–110. 10.1159/000491666 30001548

[4] Dikmen-Yildiz P, Ayers S and Phillips L. Longitudinal trajectories of post-traumatic stress disorder (PTSD) after birth and associated risk factors. J Affect Disord.2018; 229: 377–385. 10.1016/j.jad.2017.12.074 29331697

[5] Mithoefer MC, Mithoefer AT, Feduccia AA, Jerome L, Wagner M and Wymer J et al 3,4-methylenedioxymethamphetamine (MDMA)-assisted psychotherapy for post-traumatic stress disorder in military veterans, firefighters, and police officers: a randomised, double-blind, dose-response, phase 2 clinical trial. Lancet Psychiatry.2018; 5: 486–497. 10.1016/S2215-0366(18)30135-4 29728331

[6] Brown LA, Fernandez CA, Kohn R, Saldivia S and Vicente B. Pre-disaster PTSD as a moderator of the relationship between natural disaster and suicidal ideation over time. J Affect Disord.2018; 230: 7–14. 10.1016/j.jad.2017.12.096 29355729 PMC6576262

[7] Michopoulos V, Norrholm SD, Stevens JS, Glover EM, Rothbaum BO and Gillespie CF et al Dexamethasone facilitates fear extinction and safety discrimination in PTSD: A placebo-controlled, double-blind study. Psychoneuroendocrinology.2017; 83: 65–71. 10.1016/j.psyneuen.2017.05.023 28595089 PMC5524593

[8] Lai S, Wu G and Jiang Z. Glycyrrhizin Treatment Facilitates Extinction of Conditioned Fear Responses After a Single Prolonged Stress Exposure in Rats. Cellular Physiology and Biochemistry.2018; 45: 2529–2539. 10.1159/000488271 29558743

[9] Savic D, Knezevic G, Matic G and Damjanovic S. PTSD and depressive symptoms are linked to DHEAS via personality. Psychoneuroendocrinology.2018; 92: 29–33. 10.1016/j.psyneuen.2018.03.017 29621722

[10] Yehuda R. Neuroendocrine and molecular markers and PTSD. Biological Psychiatry.2016; 79: 150S 10.1016/j.biopsych.2016.03.1054

[11] Savic D, Knezevic G, Damjanovic S, Spiric Z and Matic G. The role of personality and traumatic events in cortisol levels—Where does PTSD fit in? PSYCHONEUROENDOCRINOLOGY.2012; 37: 937–947. 10.1016/j.psyneuen.2011.11.001 22133516

[12] Stalder T, Steudte-Schmiedgen S, Alexander N, Klucken T, Vater A and Wichmann S et al Stress-related and basic determinants of hair cortisol in humans: A meta-analysis. Psychoneuroendocrinology.2017; 77: 261–274. 10.1016/j.psyneuen.2016.12.017 28135674

[13] Morris MC, Compas BE and Garber J. Relations among posttraumatic stress disorder, comorbid major depression, and HPA function: A systematic review and meta-analysis. Clinical Psychology Review.2012; 32: 301–315. 10.1016/j.cpr.2012.02.002 22459791 PMC3340453

[14] Klaassens ER, Giltay EJ, Cuijpers P, van Veen T and Zitman FG. Adulthood trauma and HPA-axis functioning in healthy subjects and PTSD patients: A meta-analysis. Psychoneuroendocrinology.2012; 37: 317–331. 10.1016/j.psyneuen.2011.07.003 21802212

[15] Nijdam MJ, van Amsterdam JG, Gersons BP and Olff M. Dexamethasone-suppressed cortisol awakening response predicts treatment outcome in posttraumatic stress disorder. J Affect Disord.2015; 184: 205–8. 10.1016/j.jad.2015.05.058 26112329

[16] Sher L, Oquendo MA, Galfalvy HC, Cooper TB and Mann JJ. Age effects on cortisol levels in depressed patients with and without comorbid post-traumatic stress disorder, and healthy volunteers. J Affect Disord.2004; 82: 53–9. 10.1016/j.jad.2003.09.012 15465576

[17] Savic D, Knezevic G, Damjanovic S, Spiric Z and Matic G. The role of personality and traumatic events in cortisol levels—where does PTSD fit in? Psychoneuroendocrinology.2012; 37: 937–47. 10.1016/j.psyneuen.2011.11.001 22133516

[18] Wagner K, Couillard-Despres S, Lehner B, Brockhoff G, Rivera FJ and Blume A et al Prolactin induces MAPK signaling in neural progenitors without alleviating glucocorticoid-induced inhibition of in vitro neurogenesis. Cellular Physiology and Biochemistry.2009; 24: 397–406. 10.1159/000257432 19910680

[19] Steudte-Schmiedgen S, Stalder T, Schonfeld S, Wittchen HU, Trautmann S and Alexander N et al Hair cortisol concentrations and cortisol stress reactivity predict PTSD symptom increase after trauma exposure during military deployment. Psychoneuroendocrinology.2015; 59: 123–33. 10.1016/j.psyneuen.2015.05.007 26072152

[20] Klaassens ER, Giltay EJ, Cuijpers P, van Veen T and Zitman FG. Adulthood trauma and HPA-axis functioning in healthy subjects and PTSD patients: A meta-analysis. Psychoneuroendocrinology.2012; 37: 317–331. 10.1016/j.psyneuen.2011.07.003 21802212

[21] Meewisse M, Reitsma JB, De Vries G, Gersons BPR and Olff M. Cortisol and post-traumatic stress disorder in adults—Systematic review and meta-analysis. BRITISH JOURNAL OF PSYCHIATRY.2007; 191: 387–392. 10.1192/bjp.bp.106.024877 17978317

[22] Wahbeh H and Oken BS. Salivary Cortisol Lower in Posttraumatic Stress Disorder. Journal of Traumatic Stress.2013; 26: 241–248. 10.1002/jts.21798 23529862 PMC3818149

[23] Wingenfeld K, Whooley MA, Neylan TC, Otte C and Cohen BE. Effect of current and lifetime posttraumatic stress disorder on 24-h urinary catecholamines and cortisol: results from the Mind Your Heart Study. Psychoneuroendocrinology.2015; 52: 83–91. 10.1016/j.psyneuen.2014.10.023 25459895 PMC4297502

[24] Pan X, Wang Z, Wu X, Wen SW and Liu A. Salivary cortisol in post-traumatic stress disorder: a systematic review and meta-analysis. BMC Psychiatry.2018; 18: 324 10.1186/s12888-018-1910-9 30290789 PMC6173866

[25] Wingenfeld K, Driessen M, Adam B and Hill A. Overnight urinary cortisol release in women with borderline personality disorder depends on comorbid PTSD and depressive psychopathology. EUROPEAN PSYCHIATRY.2007; 22: 309–312. 10.1016/j.eurpsy.2006.09.002 17142011

[26] Otte C, Lenoci M, Metzler T, Yehuda R, Marmar CR and Neylan TC. Hypothalamic-pituitary-adrenal axis activity and sleep in posttraumatic stress disorder. Neuropsychopharmacology.2005; 30: 1173–1180. 10.1038/sj.npp.1300676 15714228

[27] Wheler GHT, Brandon D, Clemons A, Riley C, Kendall J and Loriaux DL et al Cortisol production rate in posttraumatic stress disorder. Journal of Clinical Endocrinology and Metabolism.2006; 91: 3486–3489. 10.1210/jc.2006-0061 16787989

[28] Murphy BE. Urinary free cortisol levels in PTSD offspring. Psychoneuroendocrinology.2003; 28: 594–5; author reply 595-6. 10.1016/s0306-4530(02)00041-0 12689615

[29] Meewisse M, Reitsma JB, De Vries G, Gersons BPR and Olff M. Cortisol and post-traumatic stress disorder in adults—Systematic review and meta-analysis. BRITISH JOURNAL OF PSYCHIATRY.2007; 191: 387–392. 10.1192/bjp.bp.106.024877 17978317

[30] Moher D, Liberati A, Tetzlaff J and Altman DG. Preferred reporting items for systematic reviews and meta-analyses: the PRISMA statement. Int J Surg.2010; 8: 336–41. 10.1016/j.ijsu.2010.02.007 20171303

[31] Samara MT, Goldberg Y, Levine SZ, Furukawa TA, Geddes JR and Cipriani A et al Initial symptom severity of bipolar I disorder and the efficacy of olanzapine: a meta-analysis of individual participant data from five placebo-controlled studies. Lancet Psychiatry.2017; 4: 859–867. 10.1016/S2215-0366(17)30331-0 28939419

[32] Chaumette B, Kebir O, Mam-Lam-Fook C, Morvan Y, Bourgin J and Godsil BP et al Salivary cortisol in early psychosis: New findings and meta-analysis. Psychoneuroendocrinology.2016; 63: 262–270. 10.1016/j.psyneuen.2015.10.007 26520686

[33] Wells G, Shea B and O'Connell J: The Newcastle-Ottawa Scale (NOS) for Assessing The Quality of Nonrandomised Studies in Meta-analyses. _journal.2014; 725286479

[34] Higgins JPT. Measuring inconsistency in meta-analyses. BMJ.2003; 327: 557–560. 10.1136/bmj.327.7414.557 12958120 PMC192859

[35] Egger M, Davey SG, Schneider M and Minder C. Bias in meta-analysis detected by a simple, graphical test. BMJ.1997; 315: 629–34. 10.1136/bmj.315.7109.629 9310563 PMC2127453

[36] Bandelow B, Baldwin D, Abelli M, Bolea-Alamanac B, Bourin M and Chamberlain SR et al Biological markers for anxiety disorders, OCD and PTSD: A consensus statement. Part II: Neurochemistry, neurophysiology and neurocognition. World Journal of Biological Psychiatry.2017; 18: 162–214. 10.1080/15622975.2016.1190867 27419272 PMC5341771

[37] Klaassens ER, Giltay EJ, Cuijpers P, van Veen T and Zitman FG. Adulthood trauma and HPA-axis functioning in healthy subjects and PTSD patients: A meta-analysis. PSYCHONEUROENDOCRINOLOGY.2012; 37: 317–331. 10.1016/j.psyneuen.2011.07.003 21802212

[38] Juster RP, Raymond C, Desrochers AB, Bourdon O, Durand N and Wan N et al Sex hormones adjust "sex-specific" reactive and diurnal cortisol profiles. Psychoneuroendocrinology.2016; 63: 282–90. 10.1016/j.psyneuen.2015.10.012 26539966

[39] Garcia NM, Walker RS and Zoellner LA. Estrogen, progesterone, and the menstrual cycle: A systematic review of fear learning, intrusive memories, and PTSD. Clin Psychol Rev.2018; 10.1016/j.cpr.2018.06.005 29945741

[40] Vieira JG, Nakamura OH and Carvalho VM. [Measurement of free urinary cortisol and cortisone using liquid chromatography associated with tandem mass spectrometry method]. Arq Bras Endocrinol Metabol.2005; 49: 291–8. doi:/S0004-27302005000200017 10.1590/s0004-27302005000200017 16184259

[41] Plenis A, Konieczna L, Oledzka I, Kowalski P and Baczek T. Simultaneous determination of urinary cortisol, cortisone and corticosterone in parachutists, depressed patients and healthy controls in view of biomedical and pharmacokinetic studies. Mol Biosyst.2011; 7: 1487–500. 10.1039/c0mb00313a 21336389

[42] Southwick SM, Axelrod SR, Wang S, Yehuda R, Morgan CR and Charney D et al Twenty-four-hour urine cortisol in combat veterans with PTSD and comorbid borderline personality disorder. J Nerv Ment Dis.2003; 191: 261–2. 10.1097/01.NMD.0000061140.93952.28 12695738

[43] Pietrzak RH, Naganawa M, Huang Y, Corsi-Travali S, Zheng MQ and Stein MB et al Association of in vivo κ-opioid receptor availability and the transdiagnostic dimensional expression of trauma-related psychopathology. JAMA Psychiatry.2014; 71: 1262–1270. 10.1001/jamapsychiatry.2014.1221 25229257

[44] Morris MC, Hellman N, Abelson JL and Rao U. Cortisol, heart rate, and blood pressure as early markers of PTSD risk: A systematic review and meta-analysis. Clinical Psychology Review.2016; 49: 79–91. 10.1016/j.cpr.2016.09.001 27623149 PMC5079809

[45] Pan X, Kaminga AC, Wen SW and Liu A. Catecholamines in Post-traumatic Stress Disorder: A Systematic Review and Meta-Analysis. Frontiers in Molecular Neuroscience.2018; 1110.3389/fnmol.2018.00450 10.3389/fnmol.2018.00450 PMC6288600 30564100

[46] Bader HN, Bierer LM, Lehrner A, Makotkine I, Daskalakis NP and Yehuda R. Maternal age at Holocaust exposure and maternal PTSD independently influence urinary cortisol levels in adult offspring. Frontiers in Endocrinology.2014; 510.3389/fendo.2014.00103 10.3389/fendo.2014.00103 PMC4081836 25071719

[47] Baker DG, West SA, Nicholson WE, Ekhator NN, Kasckow JW and Hill KK et al Serial CSF corticotropin-releasing hormone levels and adrenocortical activity in combat veterans with posttraumatic stress disorder. American Journal of Psychiatry.1999; 156: 585–588. 10.1176/ajp.156.4.585 10200738

[48] Bierer LM, Tischler L, Labinsky E, Cahill S, Foa E and Yehuda R. Clinical correlates of 24-h cortisol and norepinephrine excretion among subjects seeking treatment following the World Trade Center attacks on 9/11. Annals of the New York Academy of Sciences.2006; 1071: 514–520. 10.1196/annals.1364.055 16891610

[49] De Bellis MD, Baum AS, Birmaher B, Keshavan MS, Eccard CH and Boring AM et al Developmental traumatology Part I: Biological stress systems. BIOLOGICAL PSYCHIATRY.1999; 45: 1259–1270. 10.1016/s0006-3223(99)00044-x 10349032

[50] Delahanty DL, Raimonde AJ and Spoonster E. Initial posttraumatic urinary cortisol levels predict subsequent PTSD symptoms in motor vehicle accident victims. Biological Psychiatry.2000; 48: 940–947. 10.1016/s0006-3223(00)00896-9 11074232

[51] Lemieux AM and Coe CL. Abuse-related posttraumatic stress disorder: evidence for chronic neuroendocrine activation in women. Psychosomatic medicine.1995; 57: 105–15. 10.1097/00006842-199503000-00002 7792368

[52] Lemieux A, Coe CL and Carnes M. Symptom severity predicts degree of T cell activation in adult women following childhood maltreatment. BRAIN BEHAVIOR AND IMMUNITY.2008; 22: 994–1003. 10.1016/j.bbi.2008.02.005 18396007 PMC2532919

[53] McFarlane AC, Barton CA, Yehuda R and Wittert G. Cortisol response to acute trauma and risk of posttraumatic stress disorder. Psychoneuroendocrinology.2011; 36: 720–727. 10.1016/j.psyneuen.2010.10.007 21093988

[54] Masoudzadeh A, Modanlookordi M, Ajami A and Azizi A. Evaluation of cortisol level and cell-mediated immunity response changes in individuals with post-traumatic stress disorder as a consequence of war. European Psychiatry.2013; 28 22926353

[55] Pitman RK and Orr SP. Twenty-four hour urinary cortisol and catecholamine excretion in combat-related posttraumatic stress disorder. Biological Psychiatry.1990; 27: 245–247. 10.1016/0006-3223(90)90654-k 2294983

[56] Rasmusson AM, Lipschitz DS, Wang S, Hu S, Vojvoda D and Bremner JD et al Increased pituitary and adrenal reactivity in premenopausal women with posttraumatic stress disorder. Biological Psychiatry.2001; 50: 965–977. 10.1016/s0006-3223(01)01264-1 11750893

[57] Simeon D, Knutelska M, Yehuda R, Putnam F, Schmeidler J and Smith LM. Hypothalamic-Pituitary-Adrenal Axis Function in Dissociative Disorders, Post-Traumatic Stress Disorder, and Healthy Volunteers. Biological Psychiatry.2007; 61: 966–973. 10.1016/j.biopsych.2006.07.030 17137559 PMC2567868

[58] Wingenfeid K, Whooley MA, Neylan TC, Otte C and Cohen BE. Effect of current and lifetime posttraumatic stress disorder on 24-h urinary catecholamines and cortisol: Results from the Mind Your Heart Study. PSYCHONEUROENDOCRINOLOGY.2015; 52: 83–91. 10.1016/j.psyneuen.2014.10.023 25459895 PMC4297502

[59] Yehuda R and Bierer LM. Transgenerational transmission of cortisol and PTSD risk. Prog Brain Res.2008; 167: 121–35. 10.1016/S0079-6123(07)67009-5 18037011

[60] Yehuda R, Bierer LM, Schmeidler J, Aferiat DH, Breslau I and Dolan S. Low cortisol and risk for PTSD in adult offspring of Holocaust survivors. American Journal of Psychiatry.2000; 157: 1252–1259. 10.1176/appi.ajp.157.8.1252 10910787

[61] Yehuda R, Halligan SL and Grossman R. Childhood trauma and risk for PTSD: relationship to intergenerational effects of trauma, parental PTSD, and cortisol excretion. Development and psychopathology.2001; 13: 733–753. 10.1017/s0954579401003170 11523857

[62] Yehuda R, Morris A, Labinsky E, Zemelman S and Schmeidler J. Ten-year follow-up study of cortisol levels in aging Holocaust survivors with and without PTSD. Journal of Traumatic Stress.2007; 20: 757–761. 10.1002/jts.20228 17955524

[63] Yehuda R, Bierer LM, Sarapas C, Makotkine I, Andrew R and Seckl JR. Cortisol metabolic predictors of response to psychotherapy for symptoms of PTSD in survivors of the World Trade Center attacks on September 11, 2001. PSYCHONEUROENDOCRINOLOGY.2009; 34: 1304–1313. 10.1016/j.psyneuen.2009.03.018 19411143 PMC2785023

