# Supplementary material for: The 24-hour urinary cortisol in post-traumatic stress disorder: A meta-analysis
Source: PLoS One. 2020 Jan 9;15(1):e0227560. doi: 10.1371/journal.pone.0227560 (PMC6952249; doi:10.1371/journal.pone.0227560)
Supplement: S1 Table — Details of search strategy. (DOC) [file pone.0227560.s001.doc]

Search strategies: details of search strategy.

((PTSD[Title/Abstract] OR post-traumatic stress disorder[Title/Abstract] OR posttraumatic stress disorder[Title/Abstract])) AND (urinary free cortisol[Title/Abstract] OR urinary cortisol[Title/Abstract] OR urine cortisol[Title/Abstract] OR cortisol in urine[Title/Abstract] OR urine cortisol[Title/Abstract] OR glucocorticoids in urine[Title/Abstract] OR urine glucocorticoid[Title/Abstract] OR steroid hormones in urine[Title/Abstract] OR urine steroid[Title/Abstract] OR corticosteroids in urine[Title/Abstract] OR urine corticosteroid[Title/Abstract] OR urinary Cortisol[Title/Abstract])

56 of PubMed

TOPIC: (PTSD OR post-traumatic stress disorder OR posttraumatic stress disorder) AND TOPIC: (urinary free cortisol or urinary cortisol or urine cortisol or cortisol in urine or urine cortisol or glucocorticoids in urine or urine glucocorticoid or steroid hormones in urine or urine steroid or corticosteroids in urine or urine corticosteroid or urinary Cortisol)

373 of Web of Science

('ptsd':ab,ti OR 'post-traumatic stress disorder':ab,ti OR 'posttraumatic stress disorder':ab,ti) AND ('urinary free cortisol':ab,ti OR 'cortisol in urine':ab,ti OR 'urine cortisol':ab,ti OR 'glucocorticoids in urine':ab,ti OR 'urine glucocorticoid':ab,ti OR 'steroid hormones in urine':ab,ti OR 'urine steroid':ab,ti OR 'corticosteroids in urine':ab,ti OR 'urine corticosteroid':ab,ti OR 'urinary cortisol':ab,ti)

72 of Embase

AB (PTSD OR post-traumatic stress disorder OR posttraumatic stress disorder) AND TX (urinary free cortisol or urinary cortisol or urine cortisol or cortisol in urine or urine cortisol or glucocorticoids in urine or urine glucocorticoid or steroid hormones in urine or urine steroid or corticosteroids in urine or urine corticosteroid or urinary Cortisol)

118 of PsycARTICLES
